# Critical Care Ultrasound Competency of Fellows and Faculty in Pulmonary and Critical Care Medicine: A Nationwide Survey

**DOI:** 10.24908/pocus.v8i2.16640

**Published:** 2023-11-27

**Authors:** Mark H Adelman, Himanshu Deshwal, Deepak Pradhan

**Affiliations:** 1 Division of Pulmonary, Critical Care & Sleep Medicine, New York University Grossman School of Medicine New York, NY USA; 2 Division of Pulmonary, Critical Care, and Sleep Medicine, West Virginia University Health Sciences Center Morgantown, WV USA

**Keywords:** Point-of-Care Ultrasound (POCUS), curriculum assessment, Graduate Medical Education, point-of-care ultrasound education

## Abstract

**Purpose: **Competency assessment standards for Critical Care Ultrasonography (CCUS) for Graduate Medical Education (GME) trainees in pulmonary/critical care medicine (PCCM) fellowship programs are lacking. We sought to answer the following research questions: How are PCCM fellows and teaching faculty assessed for CCUS competency? Which CCUS teaching methods are perceived as most effective by program directors (PDs) and fellows. **Methods:** Cross-sectional, nationwide, electronic survey of PCCM PDs and fellows in accredited GME training programs. **Results:** PDs and fellows both reported the highest rates of fellow competence to use CCUS for invasive procedural guidance, but lower rates for assessment of deep vein thrombosis and abdominal organs. 54% and 90% of PDs reported never assessing fellows or teaching faculty for CCUS competency, respectively. PDs and fellows perceived hands-on workshops and directly supervised CCUS exams as more effective learning methods than unsupervised CCUS archival with subsequent review and self-directed learning. **Conclusions:** There is substantial variation in CCUS competency assessment among PCCM fellows and teaching faculty nationwide. The majority of training programs do not formally assess fellows or teaching faculty for CCUS competence. Guidelines are needed to formulate standardized competency assessment tools for PCCM fellowship programs.

## Background

Goal-directed critical care ultrasound (CCUS) has become a necessary skill set for clinicians managing critically ill patients. The Accreditation Council for Graduate Medical Education (ACGME) includes CCUS among the core procedural requirements specifically for trainees in anesthesia and emergency medicine residencies 1, 2. However, the Program Requirements for ACGME-accredited pulmonary and critical care medicine (PCCM) fellowships are less specific and more limited in scope 3. The ACGME requires that trainees demonstrate competence in the use of ultrasound to guide invasive procedures, and knowledge of imaging techniques that are used to evaluate pulmonary disease and critical illness, including ultrasound, but does not specify further which specific CCUS exams should be learned, how bedside CCUS exams should be supervised, nor how competency should be assessed. Several international pulmonary and critical care societies have since provided detailed guidelines and expectations in achieving competency in CCUS that have not been uniformly adopted across training programs^4^, ^5^, ^6^. These guidelines are based on expert consensus as there is little evidence on which to base recommendations currently.

Previous surveys of pulmonary and critical care medicine program directors demonstrated a heavy emphasis on informal bedside teaching of ultrasonography skills despite low reported levels of CCUS competency among faculty (i.e. PCCM attendings that routinely work and train fellows in the workplace setting) 7, 8. There currently exists a knowledge gap in the literature in the following areas of CCUS competency in PCCM training programs: identifying the effectiveness of commonly utilized teaching methods for CCUS; the specific assessment tools by which training programs assess for competency in CCUS; and the frequency with which these assessments occur during fellowship training 9. Additionally, little is known about methods being used to assess CCUS competency of teaching faculty in PCCM.

Better understanding of these gaps will allow for more transparency among pulmonary-critical care fellowship training programs regarding CCUS competency assessment of fellows and faculty, and allow for better standardization among programs nationwide. Our objectives were to investigate perceptions and methods utilized by fellows and teaching faculty in U.S. training programs to achieve and assess competency in CCUS.

## Methods

This study was approved by the New York University Grossman School of Medicine (NYUGSOM) Institutional Review Board (s18-00282). We conducted two cross-sectional surveys on CCUS competency from September to December of 2018: a survey of ACGME-accredited PCCM fellowship program directors or their designees, and a survey of PCCM fellows in ACGME-accredited programs. 

Surveys were designed through an iterative process of development that incorporated feedback from three groups at our institution: faculty experts in CCUS, PCCM fellowship program key clinical faculty, and senior PCCM fellows. The surveys were distributed via email to 148 PCCM fellowship program directors in the U.S. and Canada by the Association of Pulmonary and Critical Care Medicine Program Directors (APCCMPD). We asked PDs or their designee to complete the online PD survey, and forward a link to a second online survey to their fellows. We sent follow-up emails once a month for two months. 

The PD survey asked questions regarding methods used to teach CCUS to their fellows and their perceived effectiveness, perceived CCUS competency of their fellows and their teaching faculty that work with their fellows, and methods used to assess CCUS competency of their fellows and teaching faculty (see Appendix A for full PD survey). The Fellows survey asked questions regarding methods used to learn CCUS and their perceived effectiveness, their performance numbers of CCUS examinations, their perceived CCUS competency, and methods of CCUS competency assessment used by their programs (see Appendix B for full Fellows survey). Both surveys captured basic demographic information about respondents and training programs. Survey responses were anonymous and no personally identifiable information was collected. Survey study data was collected and managed using REDCap® (Research Electronic Data Capture) electronic data capture tools hosted at NYU Grossman School of Medicine 10, 11. We analyzed survey responses with IBM SPSS Statistics for Macintosh (Version 27.0, Armonk, NY) using descriptive statistics 12.

## Results

### Program Director and Fellow Surveys: Demographics

Forty program directors completed the PD survey (response rate of 27% of all programs); the total number of fellows that received survey invitations is unknown and a response rate cannot be definitively calculated but we estimated a response rate of 18%. Program and fellow demographics are described in Table 1. The vast majority were combined PCCM fellowship programs (90% on PD survey, 86% on fellow survey), academic (88%, 78%), and moderately sized (6-15 fellows, 65%, 60%). Responding fellows represented a spectrum of training years (1st year—34%, 2nd year—39%, 3rd year—23%, 4th year—5%).

**Table 1:** Fellowship Program and Fellow Demographics

-	**From PD Survey (n, %)**	**From Fellows Survey (n, %)**
**Program Type**		
Combined Pulmonary/Critical Care Medicine	36 (90%)	95 (86%)
Critical Care Medicine only	4 (10%)	16 (14%)
**Program Setting**		
Academic (University-based) Hospital	35 (88%)	87 (78%)
Community (University-affiliated) Hospital	5 (13%)	20 (18%)
Community Hospital	0 (0%)	4 (4%)
**Size of Fellowship**		
1-5 Fellows	5 (13%)	10 (9%)
6-15 Fellows	26 (65%)	67 (60%)
>15 Fellows	9 (23%)	34 (31%)
**Year of Fellowship**		
First year	-	38 (34%)
Second year		43 (39%)
Third year		25 (23%)
Fourth year or greater		5 (5%)
Values shown as number of and percentage of respondents		

### Program Director and Fellow Surveys: Perceived Faculty Competence 

The majority of PDs thought the vast majority (76-100%) of their faculty were competent to perform US-guided vascular access (62%) and US-guided drainage catheter placement (64%). The majority of PDs (59%) felt that the majority or vast majority (51%-100%) were competent in lung/pleural US. However, only a minority of PDs believed that the majority or vast majority (51%-100%) of their faculty were competent in goal-directed cardiac echo (36% of PDs), abdominal/kidney US (23%), and lower extremity DVT studies (18%).

We also performed a sub-analysis to explore if PD’s perception of faculty CCUS competence correlated with their perception of fellow CCUS competence and the strength of that correlation. Spearman’s rank correlation was computed to assess the relationship between PD’s perception of faculty competence and fellow competence for the 5 different CCUS examinations. There was a statistically significant positive correlation for goal-directed echo, r(38) = [0.436], p=0.006; and DVT studies, r(38) = [0.624], p <0.001. It was not statistically significant for US-guided vascular access (p=0.05), US-guided drainage catheter placement (p=0.271), or lung/pleural US (p=0.089).

### Methods of Teaching and Learning CCUS, and Number of CCUS Exams Performed by Fellows

Methods of teaching and learning CCUS and their perceived effectiveness are documented in Table 2. Utilized methods of teaching CCUS (PD survey) and methods of learning CCUS (Fellow survey) were similar for the two surveys--local lecture-based teaching (88%, 74% respectively), directly supervised bedside CCUS exams with feedback (85%, 74%), local hands-on workshops (73%, 69%), and self-directed learning (70%, 77%). Slightly lesser use included regional/national courses (58%, 48%), case-based didactics (65%, 52%), and unsupervised archival of images with subsequent review for teaching (60%, 51%). PD and Fellow perceptions of usefulness for these different teaching/learning methods were similar. Percentage of PDs and Fellows that perceived the different teaching/learning methods as “very” or “extremely useful” on 5-point Likert scale were: hands-on local workshops (100% of PDs, 83% of fellows), directly supervised bedside CCUS exams with feedback (90%, 94%), regional/national courses (70%, 89%), local lectures (60%, 57%), local case-based conferences (70%, 59%); unsupervised CCUS with archival of images and subsequent review (43%, 51%) and self-directed learning (30%, 49%) were rated as less useful.

**Table 2:** Methods of Teaching and Learning CCUS.

-	**From PD Survey (n, %)**	**From Fellows Survey (n, %)**	**From PD Survey (n, %)**	**From Fellows Survey (n, %)**
**Teaching and Learning Method**	Utilized by Programs to Teach CCUS (n, %)	Utilized by Fellows to Learn CCUS (n, %)	PD Perceived "Very" to "Extremely Useful" (n, %)*	Fellow Perceived "Very" to "Extremely Useful" (n, %)*
Regional/national courses	23 (58%)	53 (48%)	17 (70%)	47 (89%)
Lectures at your institution	35 (88%)	82 (74%)	19 (60%)	46 (57%)
Case-based conferences at your institution	26 (65%)	58 (52%)	19 (70%)	34 (59%)
Hands-on workshops at your institution	29 (73%)	77 (69%)	29 (100%)	63 (83%)
Directly supervised bedside CCUS exams	34 (85%)	82 (74%)	31 (90%)	77 (94%)
Unsupervised CCUS exams with saved images and subsequent reviewal	24 (60%)	57 (51%)	17 (43%)	29 (51%)
Self-directed learning	28 (70%)	85 (77%)	9 (30%)	41 (49%)
Other (blogs, US/echo tech rotation, simulation, US elective)	4 (10%)	2 (2%)	N/A	N/A
5-point Usefulness scale (1= Useless, 2= Not Very Useful, 3= Somewhat Useful, 4= Very Useful, 5= Extremely Useful); values shown as number of and percentage of respondents; *Percentage calculation--Numerator reflects number of respondents reporting "Very" or "Extremely Useful", and denominator reflects number of respondents actually utilizing the teaching/learning method.				

**Figure 1:**
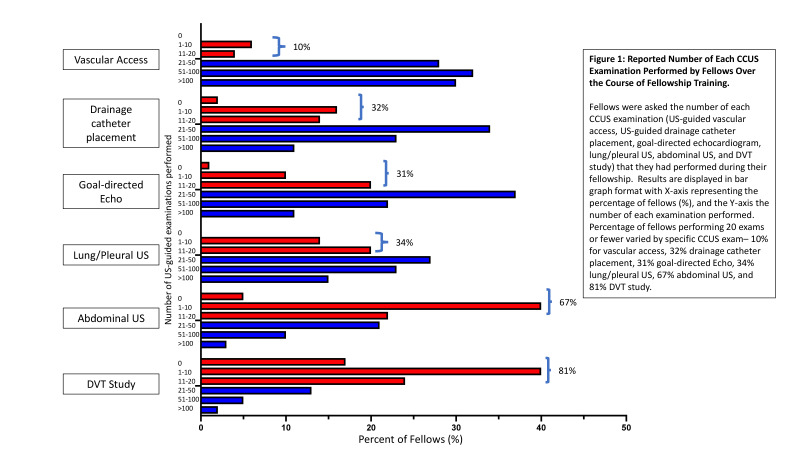
Reported Number of Each CCUS Examination Performed by Fellows Over the Course of Fellowship Training. Fellows were asked the number of each CCUS examination (US-guided vascular access, US-guided drainage catheter placement, goal-directed echocardiogram, lung/pleural US, abdominal US, and DVT study) that they had performed during their fellowship. Results are displayed in bar graph format with X-axis representing the percentage of fellows (%), and the Y-axis the number of each examination performed. Percentage of fellows performing 20 exams or fewer varied by specific CCUS exam– 10% for vascular access, 32% drainage catheter placement, 31% goal-directed Echo, 34% lung/pleural US, 67% abdominal US, and 81% DVT study.

Number of each CCUS examination performed by fellows over their fellowship is displayed in Figure 1. Percentage of fellows performing greater than 20 examinations varied by specific CCUS examination type: 90% for US-guided vascular access, 68% for US-guided drainage catheter placement, 69% for goal-directed echocardiography, 66% for lung/pleural US, 33% for abdominal US, and 19% for DVT studies.

Program Director and Fellow Surveys: Methods of Assessing Fellow and Faculty CCUS Competency 

Methods of assessing fellows for CCUS competency are detailed in Table 3. The majority of PDs (54%) report never formally assessing fellows for CCUS competency, and the majority of fellows (67%) also reported never receiving formal competency assessment. Of the programs that do assess their fellows for CCUS competency, the most used method was global assessment by expert faculty (67% on PD survey, 70% on fellow survey). Half of PDs who engage in fellow CCUS assessments reported using formal review of archived real patient images, practical exam on real patients, and use of a standardized assessment tool. However, fellows report all methods other than global assessment by faculty to be used in the minority of their programs. Of the programs that do assess their fellows for CCUS competency, the specific CCUS exams being tested varied: procedural guidance 61% use on PD survey and 46% use on fellow survey; goal-directed Echo 78% and 62% respectively; lung/pleural US 67% and 59%; abdominal/kidney US 33% and 38%, and lower extremity DVT study 50% and 35% respectively.

**Table 3:** Assessing CCUS Competency

-	**PD Survey**		**Fellow Survey**
	Fellows (n, %)	Teaching Faculty (n, %)	Fellows (n, %)
**Frequency of formal CCUS competency assessments (n= 39 for PD survey; n= 111 for Fellow survey)**			
More than once a year	7 (18%)	N/A	14 (13%)
Every year	7 (18%)	N/A	19 (17%)
Once at the end of training	4 (10%)	N/A	4 (4%)
Pre-employment	N/A	3 (8%)	N/A
More than once, but not yearly	N/A	1 (3%)	N/A
Never	21 (54%)	35 (90%)	74 (67%)
Methods of assessing CCUS competency* (n= 18 for PD survey; n= 37 for Fellow survey)			
Global assessment by expert faculty	12 (67%)	-	26 (70%)
Multiple-choice question exam	5 (28%)		12 (32%)
Formal review of archived real patient images	9 (50%)		11 (30%)
Practical exam on mannequin/simulator	5 (28%)		3 (8%)
Practical exam on standardized patient	5 (28%)		8 (22%)
Practical exam on real patient(s)	9 (50%)		8 (22%)
Use of a standardized assessment tool	9 (50%)		12 (32%)
Formal assessment by specific CCUS Exam type* (n= 18 for PD survey; n= 37 for Fellow survey)			
Procedural guidance	11 (61%)	-	17 (46%)
Goal-directed Echo	14 (78%)		23 (62%)
Lung/Pleural US	12 (67%)		22 (59%)
Abdominal/Kidney US	6 (33%)		14 (38%)
Lower extremity DVT Study	9 (50%)		13 (35%)
**Documenting CCUS competency (n= 39 for PD survey; n= 111 for Fellow survey)**			
Use of an electronic portfolio of clips/images	7 (18%)	-	7 (6%)
Required number of exams prior to fellowship completion	11 (28%)		8 (7%)
Values shown as number of and percentage of fellowship programs and fellows *Faculty numbers too low to report (given scarcity of programs assessing their faculty)			

Ninety percent of PDs reported never assessing their teaching faculty for competence in performing CCUS examinations, and 8% did so only pre-employment. Given the very low prevalence of faculty competency assessment in general, data on methods of assessing faculty competence or specific examinations being assessed were deemed too small to draw conclusions and thus not reported.

Regarding the documentation of CCUS competency, only 28% of PDs and 7% of fellows reported having a requirement for completion of a designated number of CCUS examinations prior to fellowship graduation, and only a small minority utilize an electronic portfolio to save their clips and images (18% per PDs and 6% per fellows).

### Program Director and Fellow Surveys: Perceived Fellow Competence 

etails the percentage of fellows at a given fellowship program who attain competency to independently perform basic CCUS examinations by the end of fellowship training, as well as percentage of teaching pulmonary and/or critical care faculty at a given institution currently competent to perform these same CCUS exams. 

**Table 4:** Percentage of Fellows and Teaching Faculty Perceived Competent to Independently Perform CCUS Exams.

-	**From PD Survey (n=40)**						**From Fellow Survey (n=111)**			
**CCUS Examination or Procedure**	-	Vast majority [76-100%] (n, %)	Majority [51-75%] (n, %)	Less than Half [26-50%] (n, %)	Minority [1-25%] (n, %)	None [0%] (n,%)	Perceived Competency ("Agree" or "Strongly Agree")			
							All fellows (n=111)	1st-year fellows (n= 38)	2nd-year fellows (n=43)	3rd-year or greater fellows (n=30)
Vascular access (CVL, A-line)	Fellow	37 (97%)	1 (3%)	0 (0%)	0 (0%)	0 (0%)	110 (99%)	37 (97%)	43 (100%)	30 (100%)
	Faculty	24 (62%)	7 (18%)	5 (13%)	2 (5%)	1 (3%)	-	-	-	-
Drainage catheter placement (thora, para, chest tube)	Fellow	37 (97%)	1 (3%)	0 (0%)	0 (0%)	0 (0%)	109 (98%)	36 (95%)	43 (100%)	30 (100%)
	Faculty	25 (64%)	2 (5%)	7 (18%)	5 (13%)	0 (0%)	-	-	-	-
Goal-directed Echocardiography	Fellow	18 (47%)	15 (40%)	3 (8%)	1 (3%)	1 (3%)	86 (77%)	25 (66%)	34 (79%)	27 (90%)
	Faculty	4 (10%)	10 (26%)	16 (41%)	7 (18%)	2 (5%)	-	-	-	-
Lung/Pleural US	Fellow	27 (71%)	10 (26%)	1 (3%)	0 (0%)	0 (0%)	95 (86%)	26 (68%)	39 (91%)	30 (100%)
	Faculty	9 (23%)	14 (36%)	12 (31%)	4 (10%)	0 (0%)	-	-	-	-
Abdominal and kidney US	Fellow	10 (26%)	9 (24%)	7 (18%)	10 (26%)	2 (5%)	49 (44%)	16 (42%)	20 (47%)	13 (43%)
	Faculty	2 (5%)	7 (18%)	9 (23%)	17 (44%)	4 (10%)	-	-	-	-
Lower Extremity DVT study	Fellow	14 (37%)	6 (16%)	7 (18%)	8 (21%)	3 (8%)	56 (50%)	16 (42%)	24 (56%)	16 (53%)
	Faculty	2 (5%)	5 (13%)	11 (28%)	17 (44%)	4 (10%)	-	-	-	-
Values shown as number of and percentage of respondents; Ordinal categories of "Vast majority" to "None" refer to the percent of fellows or teaching faculty competent within that training program; 5-point Agreement scale (1= Strongly disagree, 2= Disagree, 3= Neither agree nor disagree, 4= Agree, 5= Strongly agree)										

The percentage of PDs that believed the vast majority (76-100%) of their fellows attain competence varied by the specific CCUS examination type—very high perceived percentage of competence for vascular access and drainage catheter placement (97% of PDs for both), moderately perceived percentage of competence for lung/pleural US (71%), and lesser perceived percentage of competence for goal-directed echocardiography (47%), lower extremity DVT study (37%), and abdominal/kidney US (26%). Fellows’ perceptions generally agreed with PD competency perceptions for vascular access and drainage catheter placement, with fellows agreeing or strongly agreeing in their competence (99% and 98% respectively for these two procedural exams), as well as for lung/pleural exam (86% agreement with competence) and lesser perceived competence in DVT studies (50%) and abdominal/kidney US (44%). However, fellows perceived their competence higher for goal-directed echo (77% agree or strongly agree with competence) compared with PD perceptions. Breaking down fellow perceived competency by year of training, all years reported high perceived competency in US-guided vascular access (97%, 100%, 100% for 1st, 2nd, and 3rd year fellows respectively) and US-guided drainage catheter placement (95%, 100%, 100% respectively). A stepwise pattern of increasing perceived competency was seen for goal-directed Echo (66%, 79%, 90% respectively) and lung/pleural US (68%, 91%, 100%). However, rates of competence by year remained relatively flat/plateaued regardless of year of training for abdominal US (42%, 47%, 43% respectively) and DVT study (42%, 56%, 53% respectively). 

## Discussion

CCUS is a complex skill that combines cognitive knowledge along with psychomotor image acquisition skills and affective attitudes, and has become essential in daily practice for intensivists at the bedside 5. As such, in the era of competency-based medical education, it is important for medical educators to assess successful learning of this complex skill by their trainees, the ability to transfer this skill into the workplace environment, and be entrusted for independent practice 13, 14. Assessment provides transparency and a shared mental model for both teachers and learners of expectations for skills and abilities, allows for tailored learning plans dependent on skill progression, and drives learning through formative feedback 15. Unfortunately, the field of assessment in CCUS is in its relative infancy of development, with several deficiencies in standardized guidelines for longitudinal competency assessment 16. A recent systematic review found little high-quality evidence on longitudinal CCUS competence in the literature, with only 8 studies rated as “good” or “excellent” in methodologic quality and over 34 studies rated as “average” or “poor” among the 42 included studies, highlighting the need for increased and improved quality of research regarding CCUS competency 17.

Prior surveys of PCCM faculty and trainees have demonstrated heterogeneous institutional practices and methodologies to assess competency 18, 19, 20. Our study highlights important details of these methodologies and some general trends among U.S. fellowship programs including university-based, university-affiliated, and community-based hospital programs. We found general agreement between program directors and fellows regarding perceived high competency to perform CCUS for procedural guidance and lung/pleural ultrasound and perceived lower competency to perform CCUS for DVT studies and abdominal ultrasound. We saw a stepwise, incremental increase in perceived competency based on year of fellowship for goal-directed echo and lung/pleural ultrasound, high competency in procedural US throughout the years, and sustained low competence in DVT studies and abdominal ultrasound, suggesting learning curves and need for more programmatic focuses on DVT studies and abdominal ultrasound throughout all years of training. 

Interestingly, these levels of perceived competence were mirrored by fellow-reported experience with the different CCUS examinations, as most fellows reported greater experience with US-guided placement of vascular access devices or drainage catheters (e.g. thoracentesis, tube thoracostomy, paracentesis), goal-directed echocardiography and lung/pleura assessment but less abdominal and DVT ultrasound experience. About half of fellows reported performing fewer than 10 abdominal or DVT ultrasounds. ACCP/SRLF expert consensus guidelines do not specify the number of each US examination type recommended for CCUS competence 4. However, Canadian CCUS guidelines recommend at least 20 lung/pleural, 25 abdominal, and 25 vascular diagnostic ultrasound exams, and Canadian guidelines along with the European Society of Intensive Care Medicine (ESICM) guidelines recommend at least 30 TTEs for competence 21, 6. Thus, while the number of CCUS studies needed for competence is uncertain, it is clear from Figure 1 that a large number of fellows in the survey were performing fewer abdominal and DVT studies than recommended for competence. 

A survey of 67 surgical critical care fellowship program directors reported the following exams as “very important”—FAST exam (75%), central venous access (80%), transthoracic echo (47%), DVT study (3%), and abdominal US for biliary pathology (1.5%) 22. These findings further highlight the lack of valuation of DVT studies and non-procedural abdominal US examinations in critical care training programs.

Regarding CCUS competency assessment, we found in this study 54% of PDs and 67% of fellows reported that their programs never conduct formal competency assessments for CCUS. Among the programs that reported conducting formal assessments, a global assessment by expert faculty was the most common method cited by both PDs and fellows. Among those programs that do formally assess their fellows, the majority do not test specifically for competency in DVT studies or abdominal ultrasound. Additionally, there is a lack of use of archival review with feedback as well as development of ultrasound portfolios. Prior studies have documented the issue of poor faculty competence in CCUS as a barrier to training 7, 8, 9, and our study adds to our understanding the additional problem of paucity of faculty assessment in CCUS, as the vast majority (90%) of PDs reported never assessing their teaching faculty for CCUS competency. 

Given the lack of formal assessment of CCUS competency of faculty, it is uncertain what information or data PDs used to answer our survey questions on faculty competence– institutional delineation of privileges, direct observation, gestalt, or other. Of note, we saw a correlation with PD perception of faculty CCUS competency with their perception of fellow CCUS competency for goal-directed echo (moderate correlation) and DVT studies (strong correlation). This is of uncertain significance, as it could reflect causality (competence of one group leading to competence of the other group through improved educational environment and community of practice), or could represent PD’s inability to differentiate fellow from faculty competence due to lack of tangible metrics for the latter. Future studies to better understand the relationship between fellow and teaching faculty CCUS competency are warranted. 

PDs and fellows agreed on preferred methodologies for learning CCUS. Both groups reported that regional/national ultrasound courses, hands-on institutional workshops and directly supervised bedside CCUS exams were their most preferred approaches to learning CCUS, reinforcing concepts of learning through active processes. Regional CCUS courses including hands-on workshops with expert faculty have been found to be feasible and efficient in providing much needed hands-on training in CCUS 23. Although introductory CCUS courses have become ubiquitous and are attractive for time-constrained faculty development, it must be cautioned that the vast majority (93% in one study) of physicians engaging in such primer courses do not achieve sustained competence 24, and thus the focus should be on longitudinal programs for sustained competence. 

Unsupervised CCUS exams, self-directed learning and lecture-based teaching were the least favored approaches to CCUS education. Brady et al. had determined through their 20-item survey of PCCM program directors that the most common method of learning CCUS was in fact unsupervised, independent bedside learning 9, 17. Rajamani et al., in their multi-center, global study of 99 ICUs found that only 5.1% of centers provided a structured CCUS competence program for their trainees, and nearly 20.2% allowed trainees to perform unsupervised scans for clinical management without assessment of any competency. They also reported that nineteen intensivists perceived diagnostic or management errors due to misinterpretation of echocardiographic findings 25, thus cautioning against the notion that utilizing CCUS without documented competency is without potential for harm.

### Strengths and Limitations

Our survey has several strengths and limitations. Unlike previous studies, our survey included perceptions of both program directors and fellows and found a general agreement on most topics. Our findings on the perceptions of program directors with respect to the competency of clinical teaching faculty to perform CCUS have not been described previously. The major limitation of our study was the small sample size compared to all PCCM training programs which could introduce selection bias and limit the overall generalizability to all current practices. However, the consistent data from both fellows and faculty does help with overall validity of the themes that emerged from the results, and the heterogeneous distribution of academic and affiliated hospitals improves the generalizability of the findings. However, given the small sample size of this study, we would caution that the results should be viewed as intriguing but ultimately hypothesis-generating and in need of future targeted studies to expand upon this work. Also, as this is a cross-sectional survey, there could be recall biases from the PD or fellow respondents. As stated above, it is uncertain what information PDs used particularly for faculty competence given the overall lack of faculty assessment. We also relied on PDs to forward a survey link to their fellows, introducing another element of potential sampling bias to fellow responses as there was a potential gatekeeper deciding to forward or not the survey to their fellows. Thus the assumption we made is that the responding PDs and fellows likely represent the same training programs, and thus the aggregate data is representative of the same training programs. This assumption is bolstered by the Demographic information detailed in Table 1 which shows very similar PD and Fellow survey demographics (for program type, setting, and size of fellowship). However, as the data collected were anonymous, we expect the fellows to have been truthful and accurate in their responses. Lastly, given the anonymous nature of the data collection, we were able only to carry out analyses in aggregate, but unable to carry out detailed analyses of associations between PD and fellow responses from the same program.

### Future Directions

Our survey lays a foundation for future directions in the applicability and training of CCUS in clinical practice. Our exploratory analysis suggests that program director and fellow perspectives on CCUS competency overlap substantially; future surveys of PDs only might therefore be sufficient. It is also currently unknown how the COVID-19 pandemic may have impacted CCUS training despite high utilization of CCUS in intensive care units. This may help further improve our teaching practices and develop methodologies using advanced tools such as portable ultrasounds with remote access capabilities in the setting of contact precautions, virtual training and feedback sessions. It is currently unknown if CCUS training methodologies differ between CCM only programs vs PCCM programs and country-wide, survey-based studies are needed to include more CCM only programs.

## Conclusions

Our study highlights substantial heterogeneity in the CCUS teaching and competency assessment methods among ACGME-accredited PCCM programs in the United States. We found the perceptions of PDs and fellows were in general agreement with high levels of perceived competency to perform CCUS-guided procedures and lung/pleura assessment but deficiencies in the competent performance and interpretation of abdominal and lower extremity diagnostic venous ultrasonography. We also found that the majority and vast majority of programs do not assess their fellows and teaching faculty respectively for competence in CCUS, highlighting a major area of programmatic and curricular need. Our survey also demonstrates that active learning through regional and local hands-on workshops and directly supervised bedside CCUS exams were perceived as extremely useful, whereas, unsupervised CCUS exam, self-directed learning and lecture-based learning were perceived as less useful by both PDs and fellows. These findings suggest that further studies and guidelines are needed to formulate standardized competency assessment tools across all PCCM/CCM fellowship programs. 

## Disclosures

MHA had full access to all of the data in the study and takes responsibility for the integrity of the data and the accuracy of the data analysis. MHA and DP contributed substantially to the study design, data analysis and interpretation. MHA, HD and DP contributed substantially to the writing of the manuscript. The authors have no relevant conflicts of interest to disclose.

## Supplementary Material

Appendix A

Appendix A
