# Supplementary material for: Critical Care Ultrasound Competency of Fellows and Faculty in Pulmonary and Critical Care Medicine: A Nationwide Survey
Source: POCUS J. 2023 Nov 27;8(2):202–11. doi: 10.24908/pocus.v8i2.16640 (PMC10721306; doi:10.24908/pocus.v8i2.16640)
Supplement: Appendix A [file pocusj-08-16640-s001.pdf]

Ultrasound Competency Assessments in Pulmonary/Critical Care Fellowship Programs

The purpose of this study, Ultrasound Competency Assessments in Pulmonary/Critical Care Fellowship Programs: A Survey of Program Directors and Fellows, is to learn more about how pulmonary and critical care medicine fellowship programs are incorporating the use of critical care ultrasound (CCUS) in the training of fellows.

We also hope to learn more about CCUS use among faculty members that work with fellows.

Participation in this study will involve completing this one-time survey . It will take approximately 5-10 minutes of your time. All responses to this survey will be anonymous. Participation is completely voluntary and you can choose to stop answering questions at any time. Your employment, salary, and/or performance evaluation cannot be affected by your decision to participate. Record of your participation cannot be linked to your employment record.

There is no compensation offered for participation in this survey, but there is no cost to participate.

By completing this survey, you are giving your consent to be involved in this research study. If you have any questions about this study or survey, please contact mark.adelman2@nyumc.org.

Thank you!

Program Demographics

1 Your fellowship training program is for:

- ◯ Pulmonary medicine (only)
- ◯ Critical care medicine (only)
- ◯ Pulmonary and critical care medicine (combined)

2 What setting best describes your fellowship training program?

- ◯ Academic (University) Hospital
- ◯ Community Hospital
- ◯ Community Hospital (University-affiliated)

3 What is the TOTAL number of fellows in your program?

- ◯ 1-5
- ◯ 6-15
- ◯ >15

CCUS Teaching Methods for Fellows

4 What teaching methods does your program use to teach fellows how to perform CCUS? (check all that apply)

- ☐ Regional/national courses (outside your institution)
- ☐ Lectures at your institution
- ☐ Case-based conferences at your institution
- ☐ Hands-on workshop at your institution
- ☐ Directly supervised CCUS exams at the bedside
- ☐ Unsupervised CCUS exams with saved images that are reviewed with fellow later
- ☐ Self-directed learning methods (e.g. textbooks, e-books, websites)
- ☐ Other

What other methods does your program use to teach fellows how to perform CCUS? ______________________

How useful have you found these CCUS teaching methods to be for your fellows?

	Useless	Not very useful	Somewhat useful	Very useful	Extremely useful
Regional courses (outside your institution)	○	○	○	○	○
Lectures given at your institution	○	○	○	○	○
Case-based conferences at your institution	○	○	○	○	○
Hands-on workshop at your institution	○	○	○	○	○
Directly supervised CCUS exams at the bedside	○	○	○	○	○
Unsupervised CCUS exams with saved images that are reviewed with fellow later	○	○	○	○	○
Self-directed learning methods (e.g. textbooks, e-books, websites)	○	○	○	○	○

What percentage of your fellows attain competency to use ultrasound to independently perform the following procedures by the completion of fellowship training?

		0%	1-25%	26-50%	51-75%,	76-100%
5	Vascular access (e.g. CVL, a-line)	○	○	○	○	○
6	Drainage catheter placement (e.g. thoracentesis, chest tube, paracentesis)	○	○	○	○	○

What percentage of your fellows attain competency to use ultrasound to independently perform the following exams (including image acquisition and interpretation) by the completion of fellowship training?

		0%	1-25%	26-50%	51-75%	76-100%
7	Goal-directed echocardiogram	○	○	○	○	○
8	Assessment of lung and pleura	○	○	○	○	○
9	Assessment of abdomen and kidneys	○	○	○	○	○
10	Assessment for lower extremity DVT	○	○	○	○	○

CCUS Competency Assessment Methods for Fellows

11 How often does your program perform formal CCUS competency assessments for fellows?

- ◯ Never
- ◯ Once at the end of training
- ◯ Every year
- ◯ More than once a year

What methods does your program use to assess fellows for competency in the use of CCUS? (check all that apply)

- ☐ Global assessment by expert faculty
- ☐ Multiple-choice question exam
- ☐ Formal review of saved images from CCUS exams performed on real patients
- ☐ Practical exam on a mannequin/simulator
- ☐ Practical exam on a standardized patient
- ☐ Practical exam on a real patient
- ☐ Other

What other competency assessment methods does your program use?

____

For which CCUS exams do your fellows undergo a formal assessment of practical skills? (check all that apply)

- ☐ Procedure guidance
- ☐ Goal-directed echocardiogram
- ☐ Assessment of lung and pleura
- ☐ Assessment of abdomen and kidneys
- ☐ Assessment for lower extremity DVT

Does your program use a standardized evaluation tool when assessing the fellows' CCUS practical skills?

- ◯ Yes
- ◯ No

12 Are your fellows required to perform a certain number of CCUS exams prior to graduating from your training program (not including CCUS for procedural guidance only)?

- ◯ Yes
- ◯ No

How many of the following exams are your fellows required to perform?

	0	1-10	11-20	21-50	51-100	>100
Goal-directed echocardiogram	◯	◯	◯	◯	◯	◯
Assessment of lung and pleura	◯	◯	◯	◯	◯	◯
Assessment of abdomen and kidneys	◯	◯	◯	◯	◯	◯
Assessment for lower-extremity DVT	◯	◯	◯	◯	◯	◯

Do your fellows save these required exams in an electronic portfolio?

◯ Yes
◯ No

CCUS Use by Faculty

13 How many pulmonary and/or critical care attendings in your division(s) work with your fellows?

- ◯ 1-5
- ◯ 6-10
- ◯ 11-19
- ◯ 20-49
- ◯ >50

What percentage of pulmonary and/or critical care attendings that work with fellows have attained competency to independently perform and interpret the following CCUS exams?

		0%	1-25%	26-50%	51-75%	76-100%
14	Vascular access (e.g. CVL, a-line)	◯	◯	◯	◯	◯
15	Drainage catheter placement (e.g. thoracentesis, chest tube, paracentesis)	◯	◯	◯	◯	◯
16	Goal-directed echocardiogram	◯	◯	◯	◯	◯
17	Assessment of lung and pleura	◯	◯	◯	◯	◯
18	Assessment of abdomen and kidneys	◯	◯	◯	◯	◯
19	Assessment for lower extremity DVT	◯	◯	◯	◯	◯

20 How often does your division perform formal CCUS competency assessments for faculty?

- ◯ Never
- ◯ Pre-employment
- ◯ Annually
- ◯ More than once (but not every year)

What methods does your division use to assess faculty for competency in the use of CCUS? (check all that apply)

- ☐ General assessment by expert faculty
- ☐ Multiple-choice question exam
- ☐ Formal review of saved images from CCUS exams performed on real patients
- ☐ Practical exam on a mannequin/simulator
- ☐ Practical exam on a standardized patient
- ☐ Practical exam on a real patient
- ☐ Other

What other competency assessment methods does your division use?

____

For which CCUS exams do faculty undergo a formal assessment of practical skills? (check all that apply)

- ☐ Procedure guidance
- ☐ Goal-directed echocardiogram
- ☐ Assessment of lung and pleura
- ☐ Assessment of abdomen and kidneys
- ☐ Assessment for lower extremity DVT

Does your division use a standardized evaluation tool when assessing faculty members' CCUS practical skills?

- ◯ Yes
- ◯ No
