# Supplementary material for: Critical Care Ultrasound Competency of Fellows and Faculty in Pulmonary and Critical Care Medicine: A Nationwide Survey
Source: POCUS J. 2023 Nov 27;8(2):202–11. doi: 10.24908/pocus.v8i2.16640 (PMC10721306; doi:10.24908/pocus.v8i2.16640)
Supplement: Appendix A [file pocusj-08-16640-s002.pdf]

Ultrasound Competency Assessments in Pulmonary/Critical Care Fellowship Programs

The purpose of this study entitled Ultrasound Competency Assessments in Pulmonary/Critical Care Fellowship Programs: A Survey of Program Directors and Fellows, is to learn more about how pulmonary and critical care medicine fellowship programs are incorporating the use of critical care ultrasound (CCUS) in the training of fellows. Participation in this study will involve completing this one-time survey. It will take approximately 5-10 minutes of your time. All responses to this survey will be anonymous. Participation is completely voluntary and you can choose to stop answering questions at any time. Your employment, salary, and/or performance evaluation cannot be affected by your decision to participate or not. Record of your participation cannot be linked to your employment record.

There is no compensation offered for participation in this survey, but there is no cost to participate.

By completing this survey, you are giving your consent to be involved in this research study. If you have any questions about this study or survey, please contact mark.adelman2@nyumc.org

Thank you!

Demographics

1 Your fellowship training program is in:

- ◯ Pulmonary medicine (only)
- ◯ Critical care medicine (only)
- ◯ Pulmonary and critical care medicine (combined)

2 What setting best describes your fellowship training program?

- ◯ Academic (University) Hospital
- ◯ Community Hospital
- ◯ Community Hospital (University-affiliated)

3 In what year of your fellowship training are you currently?

- ◯ First Year
- ◯ Second Year
- ◯ Third Year
- ◯ Fourth Year or more

4 What is the TOTAL number of fellows in your program?

- ◯ 1-5
- ◯ 6-15
- ◯ >15

5 What methods have you used to learn how to perform CCUS during your fellowship? (check all that apply)

- ☐ Regional courses (outside your institution)
- ☐ Lectures at your institution
- ☐ Case-based conferences at your institution
- ☐ Hands-on workshop at your institution
- ☐ Directly supervised CCUS exams at the bedside
- ☐ Unsupervised CCUS exams with saved images that are reviewed with you later
- ☐ Self-directed learning methods (e.g. textbooks, e-books, websites)
- ☐ Other

What other methods have you used to learn how to perform CCUS?

____

How useful have you found these CCUS teaching methods to be?

	Useless	Not very useful	Somewhat useful	Very useful	Extremely useful
Regional courses (outside your institution)	○	○	○	○	○
Lectures given at your institution	○	○	○	○	○
Case-based conferences at your institution	○	○	○	○	○
Hands-on workshop at your institution	○	○	○	○	○
Directly supervised CCUS exams at the bedside	○	○	○	○	○
Unsupervised CCUS exams with saved images that are reviewed with you later	○	○	○	○	○
Self-directed learning methods (e.g. textbooks, e-books, websites)	○	○	○	○	○

How many times have you performed the following ultrasound-guided exams during your fellowship training?

		0	1-10	11-20	21-50	51-100	>100
6	Vascular access (e.g. CVL, a-line)	○	○	○	○	○	○
7	Drainage catheter placement (e.g. thoracentesis, chest tube, paracentesis)	○	○	○	○	○	○
8	Goal-directed echocardiogram	○	○	○	○	○	○
9	Assessment of lung and pleura	○	○	○	○	○	○
10	Assessment of abdomen and kidneys	○	○	○	○	○	○
11	Assessment for lower extremity DVT	○	○	○	○	○	○

Please rate your level of agreement with the following statement: "I feel confident in my ability to use ultrasound to independently perform the following procedures."

		Strongly disagree	Disagree	Neither agree nor disagree	Agree	Strongly agree
12	Vascular access (e.g. CVL, a-line)	○	○	○	○	○
13	Drainage catheter placement (e.g. thoracentesis, chest tube, paracentesis)	○	○	○	○	○

Please rate your level of agreement with the following statement: "I feel confident in my ability to use ultrasound to independently perform the following exams (including image acquisition and interpretation)."

		Strongly disagree	Disagree	Neither agree nor disagree	Agree	Strongly agree
14	Goal-directed echocardiogram	◯	◯	◯	◯	◯
15	Assessment of lung and pleura	◯	◯	◯	◯	◯
16	Assessment of abdomen and kidneys	◯	◯	◯	◯	◯
17	Assessment for lower extremity DVT	◯	◯	◯	◯	◯

18 How often does your program perform formal CCUS competency assessments?

- ◯ Never
- ◯ Once at the end of training
- ◯ Every year
- ◯ More than once a year

What methods does your program use to assess for CCUS competency? (check all that apply)

- ☐ General assessment by expert faculty
- ☐ Multiple-choice question exam
- ☐ Formal review of saved images from CCUS exams performed on real patients
- ☐ Practical exam on a mannequin/simulator
- ☐ Practical exam on a standardized patient
- ☐ Practical exam on a real patient
- ☐ Other

What other competency assessment methods does your program use?

____

For which CCUS exams do you undergo a formal assessment of your practical skills? (check all that apply)

- ☐ Procedure guidance
- ☐ Goal-directed echocardiogram
- ☐ Assessment of lung and pleura
- ☐ Assessment of abdomen and kidneys
- ☐ Assessment for lower extremity DVT

Does your program use a standardized evaluation tool when performing CCUS competency assessments?

- ◯ Yes
- ◯ No
- ◯ I don't know

19 Are you required to perform a certain number of CCUS exams prior to graduating from your training program (not including CCUS for procedural guidance only)?

- ◯ Yes
- ◯ No
- ◯ I don't know

How many of the following exams are you required to perform?

	0	1-10	11-20	21-50	51-100	>100
Goal-directed echocardiogram	◯	◯	◯	◯	◯	◯
Assessment of lung and pleura	◯	◯	◯	◯	◯	◯
Assessment of abdomen and kidneys	◯	◯	◯	◯	◯	◯

Assessment for lower-extremity DVT ○ ○ ○ ○ ○ ○

Do you save these required CCUS exams in an electronic portfolio?
○ Yes
○ No
